# Hemopexin as biomarkers for analyzing the biological responses associated with exposure to silica nanoparticles

**DOI:** 10.1186/1556-276X-7-555

**Published:** 2012-10-08

**Authors:** Kazuma Higashisaka, Yasuo Yoshioka, Kohei Yamashita, Yuki Morishita, Huiyan Pan, Toshinobu Ogura, Takashi Nagano, Akiyoshi Kunieda, Kazuya Nagano, Yasuhiro Abe, Haruhiko Kamada, Shin-ichi Tsunoda, Hiromi Nabeshi, Tomoaki Yoshikawa, Yasuo Tsutsumi

**Affiliations:** 1 Laboratory of Toxicology and Safety Science, Graduate School of Pharmaceutical Sciences, Osaka University, 1-6 Yamadaoka, Suita, Osaka, 565-0871, Japan; 2 Laboratory of Biopharmaceutical Research, National Institute of Biomedical Innovation, 7-6-8, Saito-Asagi, Ibaraki, Osaka, 567-0085, Japan; 3 Cancer Biology Research Center, Sanford Research/USD, 2301 E. 60th Street N, Sioux Falls, SD, 57104, USA; 4 The Center for Advanced Medical Engineering and Informatics, Osaka University, 1-6, Yamadaoka, Suita, Osaka, 565-0871, Japan; 5 Division of Foods, National Institute of Health Sciences, 1-18-1 Kamiyoga, Setagaya-ku, Tokyo, 158-8501, Japan

**Keywords:** Silica nanoparticle, Plasma proteins, Hemolysis, Biomarker

## Abstract

Practical uses of nanomaterials are rapidly spreading to a wide variety of fields. However, potential harmful effects of nanomaterials are raising concerns about their safety. Therefore, it is important that a risk assessment system is developed so that the safety of nanomaterials can be evaluated or predicted. Here, we attempted to identify novel biomarkers of nanomaterial-induced health effects by a comprehensive screen of plasma proteins using two-dimensional differential in gel electrophoresis (2D-DIGE) analysis. Initially, we used 2D-DIGE to analyze changes in the level of plasma proteins in mice after intravenous injection via tail veins of 0.8 mg/mouse silica nanoparticles with diameters of 70 nm (nSP70) or saline as controls. By quantitative image analysis, protein spots representing >2.0-fold alteration in expression were found and identified by mass spectrometry. Among these proteins, we focused on hemopexin as a potential biomarker. The levels of hemopexin in the plasma increased as the silica particle size decreased. In addition, the production of hemopexin depended on the characteristics of the nanomaterials. These results suggested that hemopexin could be an additional biomarker for analyzing the biological responses associated with exposure to silica nanoparticles. We believe that this study will contribute to the development of biomarkers to ensure the safety of silica nanoparticles.

## Background

Nanomaterials with particle sizes below 100 nm display unique properties compared to conventional materials with a submicron size. Various types of nanomaterials have been designed and produced for consumer and industrial applications such as medicine, cosmetics, and food [1,2]. As the use of nanomaterials increases, there is a growing need to ensure their safety because their unique properties might be associated with undesirable biological interactions [3,4]. However, current knowledge of the potential risk of nanomaterials is considered insufficient. Therefore, to facilitate the development of nanomaterials as a safe and usable product, it is important to develop guidelines for evaluation of their safety and efficacy.

Silica nanoparticles have been widely used in many consumer products such as cosmetics, food, and medicine because of their useful properties, including straightforward synthesis, relatively low cost, easy separation, and easy surface modification [5,6]. However, recent studies have found that silica nanoparticles induce substantial lung inflammation and are cytotoxic to various cell types [7,8]. Furthermore, our group showed that silica nanoparticles penetrate the skin and produce systemic exposure after topical application [9]. These findings underscore the need to examine biological effects after systemic exposure to silica nanoparticles. Our group also demonstrated that intravenous injection of silica nanoparticles with a diameter of 70 nm into mice might induce severe liver damage [9-12] and pregnancy complications such as resorption and fetal growth restriction [13]. We also showed that these pregnancy complications can be suppressed by amino or carboxyl group surface modification [11,13].

The development of safe nanomaterials requires not only an evaluation of safety, but also the ability to predict their biological effects. Molecular biomarkers constitute an objective indicator for correlating against various physiological conditions or variation of disease state [14,15]. Biomarker studies have the potential to provide valuable information to identify early biological events associated with the adverse health effects of engineered nanomaterials in a development stage more easily and rapidly [16]. Studies of biomarkers for nanomaterials have barely advanced, but it is envisaged that a biomarker profile for exposure to nanomaterials would represent the unity of local and systemic physiological responses induced as a result of exposure. Therefore, there is a need to identify and evaluate biomarkers for nanomaterials that would be suitable for predicting the potential toxicity of nanomaterials as well as to facilitate the development of nanomaterials that are safe. In this regard, our previous study used sodium dodecyl sulfate-polyacrylamide gel electrophoresis (SDS-PAGE) analysis to show that the acute-phase proteins, haptoglobin, C-reactive protein, and serum amyloid A (SAA), can act as useful biomarkers for analyzing the risk of exposure to nanomaterials and their associated toxicity [17]. However, SDS-PAGE analysis has limited capacity for a comprehensive screen for biomarkers because it is based only on differences in molecular weight of proteins. Proteomics-based analyses such as two-dimensional (2D) gel separation and mass spectrometry are more suitable approaches for such a comprehensive study. Here, we performed a screen for biomarkers of nanomaterials using two-dimensional differential in gel electrophoresis (2D-DIGE), which is a gel-based approach like SDS-PAGE but separates proteins on the basis of their molecular weight and isoelectric point. We used this approach to identify hemopexin as a potential biomarker for predicting the biological effects induced by silica nanoparticles.

## Methods

### Materials

Silica particles were purchased from Micromod Partikeltechnologie (Rostock/Warnemünde, Germany). Silica particles with diameters of 70, 300, and 1,000 nm (nSP70, nSP300, and mSP1000, respectively) and nSP70 with surface carboxyl and amino groups (nSP70-C and nSP70-N, respectively), were used in this study. The silica particles were suspended in saline, sonicated for 5 min, and vortexed for 1 min prior to use.

### Animals

Female BALB/c mice were purchased from Nippon SLC, Inc. (Shizuoka, Japan) and were used at 6 to 8 weeks of age. The mice were housed in a ventilated animal room maintained at 20 ± 2°C with a 12-h light/12-h dark cycle. The mice had free access to water and forage (FR-2, Funabashi farm, Chiba, Japan). All of the animal experimental procedures used in this study were performed in accordance with the Osaka University and National Institute of Biomedical Innovation guidelines for the welfare of animals.

### 2D-DIGE analysis

The BALB/c mice were treated intravenously with 0.8 mg/mouse nSP70 or saline. After 24 h, blood samples were collected, and plasma was harvested by centrifuging blood at 13,800×*g* for 15 min. ProteoPrep (Sigma-Aldrich, Saint Louis, MO, USA) was used to remove albumin and immunoglobulin from the plasma according to the manufacturer's instructions. Plasma proteins were purified from the plasma of the nSP70- or saline-treated mice using a 2D-Clean up Kit (GE Healthcare Biosciences, Piscataway, NJ, USA) and were labeled at the ratio of 50 μg proteins:400 pmol Cy3 or Cy5 protein-labeling dye (GE Healthcare Biosciences) in dimethylformamide according to the manufacturer's protocol. Briefly, 50 μg of each labeled sample was mixed with rehydration buffer (7 M urea, 2 M thiourea, 4% 3-(3-cholamidepropyl) dimethylammonio-1-propanesulphonate, 2% dithiothreitol, 2% Pharmalyte; GE Healthcare Biosciences) and applied to a 24-cm immobilized pH gradient gel strip (immobilized pH gradient (IPG) strip pH 4 to 7 NL) for separation in the first dimension. Samples for the spot picking gel were prepared without labeling by Cy dyes. For the second-dimension separation, the IPG strips were applied to SDS-PAGE gels (10% polyacrylamide and 2.7% *N*,*N*′-diallyltartardiamide gels). After electrophoresis, the gels were scanned with a laser fluoroimager (Typhoon Trio, GE Healthcare Biosciences). The spot picking gel was scanned after staining with Deep Purple Total Protein Stain (GE Healthcare Biosciences). Quantitative analysis of protein spots was carried out with Decyder-DIA software (GE Healthcare Biosciences). Protein spots representing greater than twofold alteration in expression were picked using an Ettan Spot Picker (GE Healthcare Biosciences).

### In-gel tryptic digestion

The gel pieces were destained with 50% acetonitrile (ACN)/25 mM NH_4_HCO_3_ for 10 min, dehydrated with 100% ACN for 10 min, and then dried using a centrifugal concentrator (TOMY SEIKO, Tokyo, Japan). Next, 8 μl of 20 μl/ml trypsin solution (Promega, Madison, WI, USA) diluted fivefold in 50 mM NH_4_HCO_3_ was added to each gel piece, which was then incubated overnight at 37°C. We used three solutions to extract the resulting peptide mixtures from the gel pieces. First, 50 μl of 50% (*v*/*v*) ACN in 0.1% aqueous trifluoroacetic acid (TFA) was added to the gel pieces, which were then sonicated for 30 min. Next, we collected the solution and added 80% (*v*/*v*) ACN in 0.1% TFA. Finally, 100% ACN was added for the last extraction. The peptide solutions were dried and resuspended in 10 μl of 0.1% formic acid. The resulting peptide mixture was then analyzed by nano-flow liquid chromatography/tandem mass spectrometry (maXis, Bruker Daltonik GmbH, Bremen, Germany). The Mascot search engine (Matrix Science Inc., Boston, MA, USA) was initially used to query the entire theoretical tryptic peptide as well as the Swiss-Prot protein sequence database.

### Measurement of hemopexin

The BALB/c mice were treated intravenously with 0.8 mg/mouse of the silica particles nSP70, nSP300, mSP1000, nSP70-C, and nSP70-N or with saline. Blood samples were collected at 2, 6, 24, 48, and 72 h after treatment. For assessment of the sensitivity of hemopexin levels to the concentration of silica particles, the BALB/c mice were treated intravenously with 0.05, 0.2, or 0.8 mg/mouse nSP70. After 24 h, blood samples were collected, and plasma was harvested by centrifuging blood at 13,800×*g* for 15 min. Plasma levels of hemopexin were measured using a commercial enzyme-linked immunosorbent assay (ELISA) kit (Life Diagnostics, West Chester, PA, USA), according to the manufacturer's instructions.

### Plasma biochemistry

The BALB/c mice were treated intravenously with 0.8 mg/mouse nSP70 or saline. After 2, 6, 24, and 48 h, blood samples were collected, and plasma was harvested by centrifuging blood at 13,800×*g* for 15 min. Total hemoglobin and heme in the blood of the nSP70-treated mice were determined by BioAssay Systems QuantiChrom^TM^ Assay Kits (BioAssay Systems, Hayward, CA, USA). Also, plasma levels of total bilirubin (TBIL) and direct bilirubin (DBIL) were measured by a biochemical auto analyzer, FUJI DRI-CHEM 7000 (Fujifilm, Tokyo, Japan), and the level of indirect bilirubin was calculated from these values.

### Statistical analysis

All results are expressed as means ± standard error of the mean (SEM). Statistical comparisons between groups were performed by one-way analysis of variance (ANOVA) with the Bonferroni test.

## Results

### Characteristics of silica particles

Silica particles are well suited for studying the influence of nanomaterial size on biodistribution and various biological effects because they show much better dispersibility in aqueous solutions than most other nanomaterials [18]. We used three different-sized silica particles with diameters between 70 and 1,000 nm (nSP70, nSP300, and mSP1000) and nSP70 with carboxyl (nSP70-C) and amino (nSP70-N) surface functional groups. As we have described previously [9,10,13], all silica nanoparticles were confirmed by transmission electron microscopy to be smooth-surfaced spheres. The hydrodynamic diameters of nSP70, nSP300, and mSP1000 were 65, 322, and 1,140 nm, respectively, and their zeta potentials or overall surface potentials were −53, −62, and −67 mV, respectively. For nSP70-C and nSP70-N, the hydrodynamic diameters were 70 and 72 nm, respectively, and their zeta potentials were −76 and −29 mV, respectively. These results indicate that the carboxyl and amino surface modifications altered the surface charge of the particles. The size distribution spectrum of each set of silica particles showed a single peak, and the measured hydrodynamic diameter corresponded almost precisely to the primary particle size of each set of silica particles. These results indicate that the silica particles used in this study were well dispersed in solution.

### 2D-DIGE analysis and identification of differentially expressed proteins

**Figure 1:**
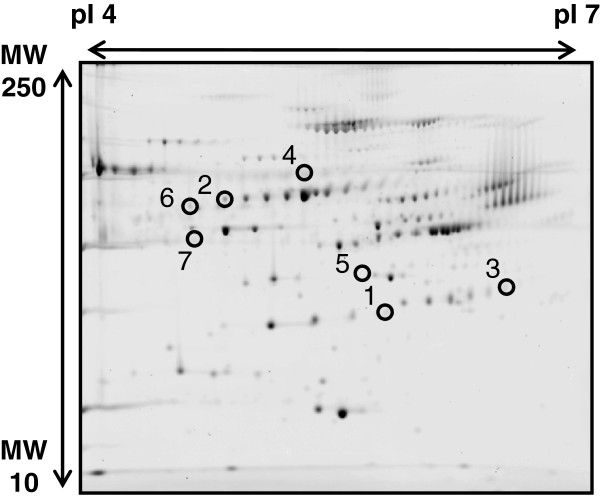
**2D-DIGE image of fluorescent-labeled proteins.** Proteins from the plasma after treatment with saline or nSP70 were labeled with Cy3 and Cy5, respectively, and used for performing 2D electrophoresis.

To identify protein biomarkers of nanomaterials in mice, we analyzed changes in the levels of plasma proteins following treatment with nSP70 by using 2D-DIGE. Plasma proteins isolated after treatment with saline or 0.8 mg/mouse nSP70 were labeled with Cy3 and Cy5, respectively, and were used for 2D-DIGE analysis. The reason why we chose the dose of silica particles for treatment is that none of the silica particles induced any significant changes in the levels of aspartate aminotransferase (AST), alanine aminotransferase (ALT), and blood urea nitrogen and that all parameters remained within the physiological range, as we have previously reported [17]. Quantitative image analysis revealed 59 spots showing increased protein levels and 23 spots showing decreased protein levels in the plasma of nSP70-treated mice compared with controls. We selected a total of eight candidate spots showing the highest increases and decreases in protein expression levels. Then, liquid chromatography/time-of-flight/mass spectrometry (LC/TOF/MS) analysis of the spots subsequently identified seven different proteins (Figure 1). Among these proteins, haptoglobin, hemopexin, and alpha-1-acid glycoprotein 1, which are acute-phase proteins, displayed increased expression in the plasma of nSP70-treated mice. Four proteins, including inter-alpha-trypsin inhibitor, complement C4-B, Cullin-4A, and serotransferrin, displayed decreased expression in the plasma of nSP70-treated mice (Table 1). These findings are consistent with the results of our previous study identifying haptoglobin, showing the highest level in this study, as a candidate biomarker using SDS-PAGE analysis. We selected a candidate biomarker from among the proteins which displayed increased expression at first. However, in the future, there is a need to identify various biomarkers by evaluating candidate proteins which displayed not only increased expression but also decreased expression to improve the accuracy for predicting the biological effects induced by nanomaterials. Here, we focused on hemopexin, which showed the second highest expression level among the identified candidate biomarkers after haptoglobin. 

**Table 1:** Identification of candidate proteins as biomarkers

**Spot**	**Protein name**	**Accession number**	**MW (kD)**	**pI**	**Expression ratio (nSP70/saline) (fold)**
1	Haptoglobin	[Swiss-Prot:Q61646]	38.75	5.88	375.44
2	Hemopexin	[Swiss-Prot:Q91X72]	51.34	7.92	3.25
3	Alpha-1-acid glycoprotein 1	[Swiss-Prot:Q60590]	23.89	5.58	3.05
4	Inter-alpha-trypsin inhibitor	[Swiss-Prot:Q61703]	105.93	6.82	0.40
5	Complement C4-B	[Swiss-Prot:P01029]	192.89	7.38	0.39
6	Cullin-4A	[Swiss-Prot:Q3TCH7]	87.75	8.53	0.37
7	Serotransferrin	[Swiss-Prot:Q921l1]	76.72	6.94	0.30

The differentially expressed spots were identified by LC/TOF/MS. pI, isoelectric point.

### Plasma hemopexin levels after treatment with silica particles

**Figure 2:**
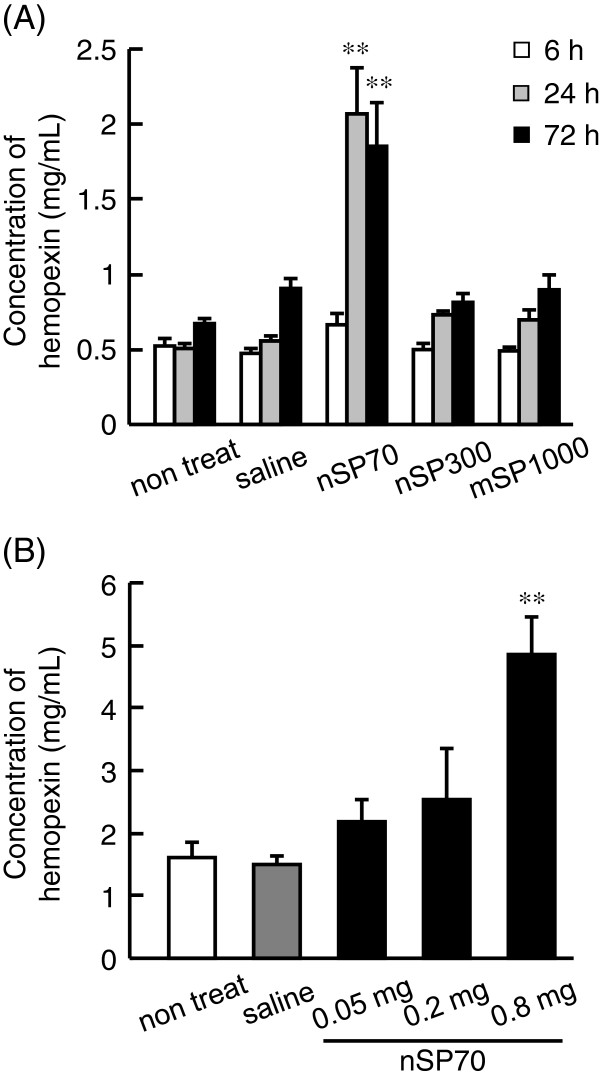
**Plasma levels of hemopexin after treatment with silica particles.** BALB/c mice were treated intravenously with 0.8 mg/mouse nSP70, nSP300, or mSP1000. (**A**) After 6, 24, and 72 h, the plasma levels of hemopexin in the mice were determined by ELISA. (**B**) BALB/c mice were treated intravenously with 0.05, 0.2, or 0.8 mg/mouse nSP70. After 24 h, blood samples were collected. The plasma levels of hemopexin in treated mice were determined by ELISA. Data are presented as mean ± SEM (*n* = 5; Double asterisks denote *P* < 0.01 versus the value for saline-treated group by ANOVA).

Hemopexin is known as an acute-phase protein, mainly synthesized in the liver [19]. To assess the potential of hemopexin as a biomarker, we examined whether there were time-dependent changes in the plasma levels of hemopexin levels after treatment with different-sized silica particles. The BALB/c mice were treated intravenously with 0.8 mg/mouse nSP70, nSP300, or mSP1000. After 6, 24, or 72 h, we examined the plasma levels of hemopexin by ELISA. We observed no changes in the plasma levels of hemopexin in mice treated with nSP300 or mSP1000 over the time course of the experiment. However, mice treated with nSP70 showed an increase in plasma levels of hemopexin at 24 h after treatment, and the plasma level of hemopexin in nSP70-treated mice remained significantly higher than that of controls at 72 h after treatment (Figure 2A). These results indicate that the smaller the particle size, the greater the increase in plasma levels of hemopexin induced by silica particles. We then assessed the sensitivity of hemopexin induction to lower concentrations of silica particles. The BALB/c mice were treated intravenously with 0.05, 0.2, or 0.8 mg/mouse nSP70. After 24 h, we examined the plasma levels of hemopexin by ELISA and found that the plasma level of hemopexin increased in a dose-dependent manner (Figure 2B). These results indicate that the level of induction of hemopexin is dependent on the concentration of silica particles. Taken together, these findings highlight the potential of hemopexin as a valuable biomarker for analyzing the risk and toxicity of exposure to silica nanoparticles. Now, to evaluate the sensitivity of hemopexin to serve as a biomarker of a more realistic exposure, we have assessed the response of hemopexin to silica nanoparticles introduced via different routes. 

### Hemolytic activity of silica nanoparticles

**Figure 3:**
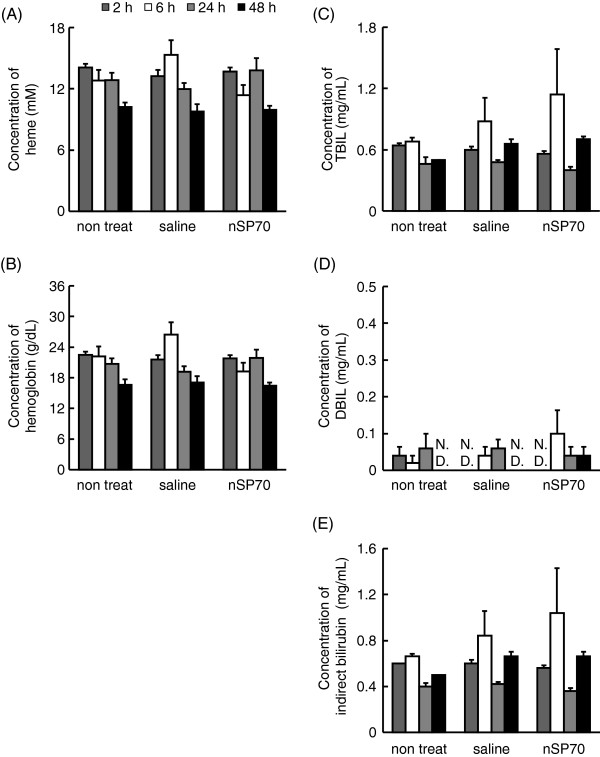
**Hemolytic activity of silica nanoparticles.** BALB/c mice were treated intravenously with 0.8 mg/mouse nSP70. After 2, 6, 24, and 48 h, we examined the level of (**A**) total heme and (**B**) hemoglobin in the blood of treated mice. The levels of (**C**) TBIL and (**D**) DBIL were measured, and (**E**) the level of indirect bilirubin was calculated from these values.

Hemopexin is a heme-binding plasma glycoprotein that forms the second line of defense against hemoglobin-mediated oxidative damage during intravascular hemolysis [20]. Intravascular hemolysis causes the release of massive amounts of hemoglobin and heme into the plasma, where they are rapidly bound by haptoglobin and hemopexin, respectively [21]. Because nSP70 induced an increase in the plasma levels of haptoglobin and hemopexin in mice, we investigated the possibility that silica nanoparticles could induce hemolytic activity. We assessed plasma levels of heme and hemoglobin 2, 6, 24, or 48 h after treatment of the BALB/c mice intravenously with 0.8 mg/mouse of nSP70. At this dose, nSP70 did not induce any significant increases in the plasma levels of heme (Figure 3A) or hemoglobin (Figure 3B) at all time points. 

During hemolysis, hemoglobin is released into the plasma from damaged red blood cells and leads to an increase in plasma levels of indirect bilirubin [22-24]. Thus, although nSP70 did not induce a significant increase in the plasma levels of heme or hemoglobin, we investigated whether nSP70 induced hemolytic activity that resulted in an increased plasma level of indirect bilirubin. Following treatment of mice with nSP70, we measured plasma levels of TBIL and DBIL and calculated the level of indirect bilirubin from these values. We found no changes in the plasma levels of TBIL (Figure 3C), DBIL (Figure 3D), or indirect bilirubin (Figure 3E) over the time course of the experiment. Taken together, these results clearly show that nSP70 does not induce hemolytic activity in mice under these conditions.

### Surface modification of nSP70

**Figure 4:**
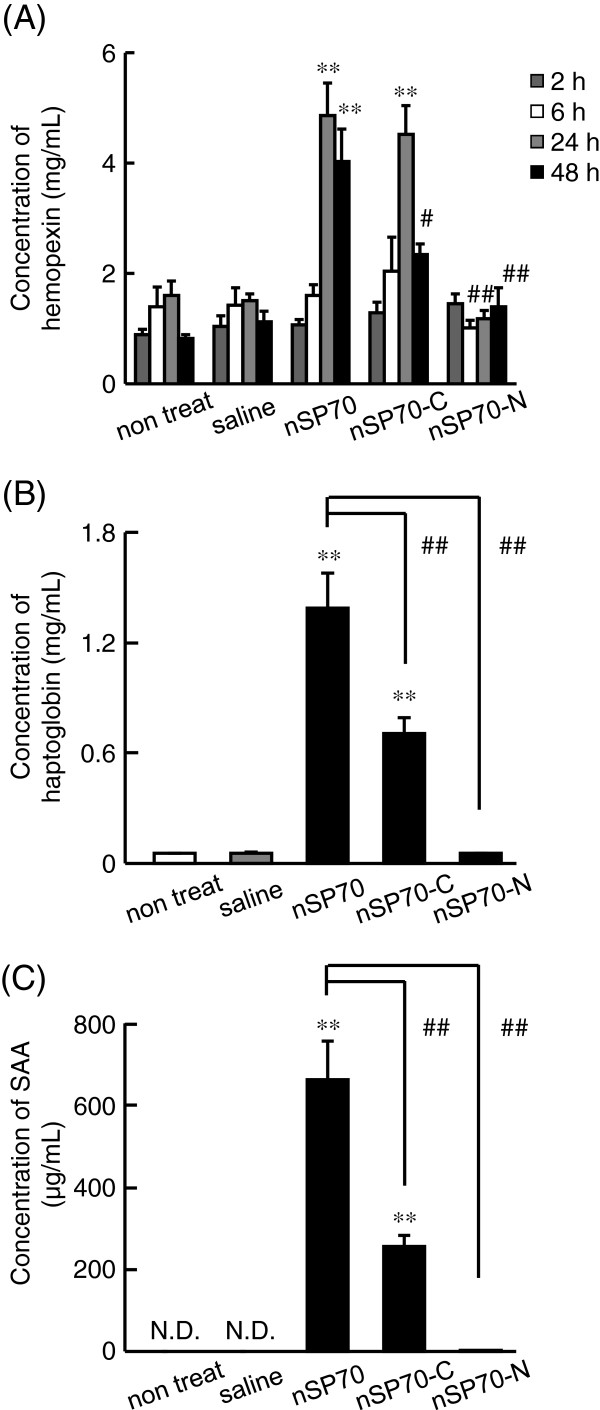
**Responses of hemopexin by the exposure to surface modified nSP70.** BALB/c mice were treated intravenously with nSP70 modified with amino (nSP70-N) or carboxyl (nSP70-C) groups at 0.8 mg/mouse. (**A**) After 2, 6, 24, and 48 h, the levels of hemopexin in the plasma of treated mice were examined by ELISA. The plasma levels of (**B**) haptoglobin and (**C**) SAA were determined by ELISA after 24 h. Data are presented as mean ± SEM. (*n* = 5; Double asterisks denote *P* < 0.01 versus the value for the saline-treated group by ANOVA, single hash denotes *P* < 0.05, and double hash denote *P* < 0.01 versus the value for the nSP70-treated group by ANOVA).

Previously, we demonstrated that surface properties of silica nanoparticles play an important role in determining their safety [11,13]. For instance, our group showed that surface modification of nSP70 with amine or carboxyl groups altered the intracellular distribution of the nanoparticles, had an effect on cell proliferation [11], and suppressed toxic biological effects of silica particles such as inflammatory responses. To assess whether hemopexin could predict the strength of toxicity induced by silica nanoparticles, we examined the plasma levels of hemopexin in the mice after administration of nSP70 with amino or carboxyl group surface modifications. The BALB/c mice were treated intravenously with 0.8 mg/mouse of nSP70, nSP70-C, or nSP70-N. After 2, 6, 24, or 48 h, we examined the plasma levels of hemopexin by ELISA. The mice treated with nSP70, nSP70-C, and nSP70-N did not show any elevated level of hemopexin at 2 or 6 h. At 24 h, the plasma level of hemopexin in mice treated with nSP70-N was significantly lower than that in mice treated with unmodified nSP70. On the other hand, the plasma level of hemopexin in mice treated with nSP70-C was similar to that in nSP70-treated mice and significantly higher than that in saline-treated mice (Figure 4A). At the same time, the plasma levels of haptoglobin (Figure 4B) and SAA (Figure 4C) in mice treated with nSP70-C were significantly lower than those in nSP70-treated mice, which is consistent with our previously reported results [17]. These results indicate that there are differences in the mechanisms underlying the production of hemopexin and other acute-phase proteins induced by nSP70-C. 

## Discussion

By using biomarkers, we are able to predict not only the present disease and clinical condition, but also the risk of acquiring disease in the future. Therefore, it is necessary to progress studies of biomarkers for nanomaterials because very little information is available on the biological effects of nanomaterials. Here, we used 2D-DIGE analysis to perform a comprehensive screen of plasma proteins to identify protein biomarkers of nanomaterials. We identified hemopexin (Table 1) as a useful biomarker for analyzing the biological responses associated with exposure to silica nanoparticles. Because 2D-DIGE is a proteomic method, this approach has potential to uncover the as yet unknown biological effects of nanomaterials.

On the other hand, an inherent disadvantage of 2D electrophoresis is the poor resolution of hydrophobic proteins [25,26]. Hence, it is likely that integral membrane proteins are strongly underrepresented. Isobaric tags for relative and absolute quantification or iTRAQ is a comprehensive gel-free quantitative proteomic method based on mass spectrometry [27,28]. This approach can be used to generate proteomic profiles that reflect the pathological state of organs and aid in the early detection of diseases [29,30]. We envisage that a combination of comprehensive proteomic methods would help to identify potential toxicities of nanomaterials during their development and contribute to the establishment of strategies to ensure their safety.

Silica materials with amorphous particle morphology are known to cause the hemolysis of mammalian red blood cells [31,32]. For this reason, we investigated the possibility that silica nanoparticles could induce hemolytic activity. While the exact mechanism is still under investigation, most reports agree that the hemolytic activity of silica particles is related to surface characteristics such as area and curvature [33-35]. However, we found that nSP70 at 0.8 mg/mouse does not induce hemolytic activity through elevation of haptoglobin and hemopexin (Figure 3). We are continuing investigations to understand the biological mechanisms associated with the elevation of hemopexin after exposure to silica nanoparticles.

We then examined the effects of surface modification of silica nanoparticles on the production of hemopexin. Compared to controls, hemopexin was not induced by nSP70-N but was induced to a significantly higher level by nSP70-C, which was a similar level to that induced by unmodified nSP70 (Figure 4A). On the other hand, the plasma levels of haptoglobin (Figure 4B) and SAA (Figure 4C) in mice treated with nSP70-C were significantly lower than those in mice treated with nSP70, as reported previously [17]. These results suggest that the production of acute-phase proteins depends on the characteristics of the nanomaterials and that nSP70-C induces some biological effects associated with the elevation of hemopexin. Increased hemopexin levels have been found in patients with diabetes mellitus and are associated with some malignancies, such as malignant melanoma and breast cancer [20,36,37]. Elevated hemopexin levels have also been found in inflammatory psychiatric disorders, such as major depression, schizophrenia, and mania [38]. Taken together, these findings suggest that hemopexin might be associated with these diseases so it is possible that the induced elevation of hemopexin by both nSP70 and nSP70-C is related to the induction of inflammatory responses. Therefore, we are currently analyzing not only the mechanisms underlying the differences in production of hemopexin, haptoglobin, and SAA induced by nSP70-C and nSP70, but also the relationship. It could be speculated that there are differences between the fates of the injected nSP70 and nSP70-C. Therefore, there is a need to evaluate the distribution or accumulation of the injected nanoparticles in the liver where acute-phase proteins are known to be produced. An understanding of these mechanisms will advance the use of biomarkers for different purposes and improve the predictive value of these biomarkers.

Hemopexin is one of the acute-phase proteins released from the liver, and its production is known to be regulated by cytokines. For instance, interleukin (IL)-6 and IL-22 induce the hepatic production of circulating SAA [39,40]. Furthermore, it is conceivable that instead of inflammatory cytokines, small silica particles act directly on the liver to induce the release of acute-phase proteins. However, nSP70, at this dose, did not induce any significant elevation of liver injury or dysfunction markers, such as ALT or AST. Therefore, it is unclear why nanomaterials induce the production of acute-phase proteins. We are currently analyzing the detailed mechanism by which silica particles induce acute-phase proteins.

## Conclusions

We demonstrated that 2D-DIGE analysis is a useful approach for identifying novel biomarkers of nanomaterials. Using this approach, we identified hemopexin as a useful biomarker and showed here that hemopexin can act as a useful biomarker for analyzing the biological responses associated with exposure to silica nanoparticles. We believe that this study will contribute to the development of biomarkers for ensuring the safety of silica nanoparticles.

## Abbreviations

2D: Two-dimensional; 2D-DIGE: Two-dimensional differential in gel electrophoresis; ACN: Acetonitrile; ALT: Alanine amino transferase; AST: Aspartate amino transferase; DBIL: Direct bilirubin; ELISA: Enzyme-linked immunosorbent assay; IL: Interleukin; IPG: Immobilized pH gradient; iTRAQ: Isobaric tags for relative and absolute quantification; LC/TOF/MS: Liquid chromatography/time-of-flight/mass spectrometry; SAA: Serum amyloid A; SDS-PAGE: Sodium dodecyl sulfate-polyacrylamide gel electrophoresis; TBIL: Total bilirubin; TFA: Trifluoroacetic acid.

## Competing interests

The authors declare that they have no competing interests.

## Authors’ contributions

KH and YY designed the study. KH, KY, YM, HP, TO, TN, and AK performed the experiments. KH and YY collected and analyzed the data. KH and YY wrote the manuscript. KN, YA, HK, ST, HN, and TY gave technical support and conceptual advice. YT supervised all of the projects. All authors discussed the results and commented on the manuscript. All authors read and approved the final manuscript.
